# Comprehensive and comparative studies on nanocytotoxicity of glyceryl monooleate- and phytantriol-based lipid liquid crystalline nanoparticles

**DOI:** 10.1186/s12951-021-00913-5

**Published:** 2021-06-03

**Authors:** Jakub Jagielski, Łucja Przysiecka, Dorota Flak, Magdalena Diak, Zuzanna Pietralik-Molińska, Maciej Kozak, Stefan Jurga, Grzegorz Nowaczyk

**Affiliations:** 1 NanoBioMedical Centre, Adam Mickiewicz University, Wszechnicy Piastowskiej 3, 61-614 Poznań, Poland; 2 Department of Macromolecular Physics, Faculty of Physics, Adam Mickiewicz University, Uniwersytetu Poznańskiego 2, 61-614 Poznań, Poland

**Keywords:** Cubosomes, Cytotoxicity, Genotoxicity, Cytoskeleton integrity, Reactive oxygen species generation, Cellular internalization

## Abstract

**Background:**

Lipid liquid crystalline nanoparticles (LLCNPs) emerge as a suitable system for drug and contrast agent delivery. In this regard due to their unique properties, they offer a solubility of a variety of active pharmaceutics with different polarities increasing their stability and the possibility of controlled delivery. Nevertheless, the most crucial aspect underlying the application of LLCNPs for drug or contrast agent delivery is the unequivocal assessment of their biocompatibility, including cytotoxicity, genotoxicity, and related aspects. Although studies regarding the cytotoxicity of LLCNPs prepared from various lipids and surfactants were conducted, the actual mechanism and its impact on the cells (both cancer and normal) are not entirely comprehended. Therefore, in this study, LLCNPs colloidal formulations were prepared from two most popular structure-forming lipids, i.e., glyceryl monooleate (GMO) and phytantriol (PHT) with different lipid content of 2 and 20 w/w%, and the surfactant Pluronic F-127 using the top-down approach for further comparison of their properties. Prepared formulations were subjected to physicochemical characterization and followed with in-depth biological characterization, which included cyto- and genotoxicity towards cervical cancer cells (HeLa) and human fibroblast cells (MSU 1.1), the evaluation of cytoskeleton integrity, intracellular reactive oxygen species (ROS) generation upon treatment with prepared LLCNPs and finally the identification of internalization pathways.

**Results:**

Results denote the higher cytotoxicity of PHT-based nanoparticles on both cell lines on monolayers as well as cellular spheroids, what is in accordance with evaluation of ROS activity level and cytoskeleton integrity. Detected level of ROS in cells upon the treatment with LLCNPs indicates their insignificant contribution to the cellular redox balance for most concentrations, however distinct for GMO- and PHT-based LLCNPs. The disintegration of cytoskeleton after administration of LLCNPs implies the relation between LLCNPs and F-actin filaments. Additionally, the expression of four genes involved in DNA damage and important metabolic processes was analyzed, indicating concentration–dependent differences between PHT- and GMO-based LLCNPs.

**Conclusions:**

Overall, GMO-based LLCNPs emerge as potentially more viable candidates for drug delivery systems as their impact on cells is not as deleterious as PHT-based as well as they were efficiently internalized by cell monolayers and 3D spheroids.

**Graphic Abstract:**

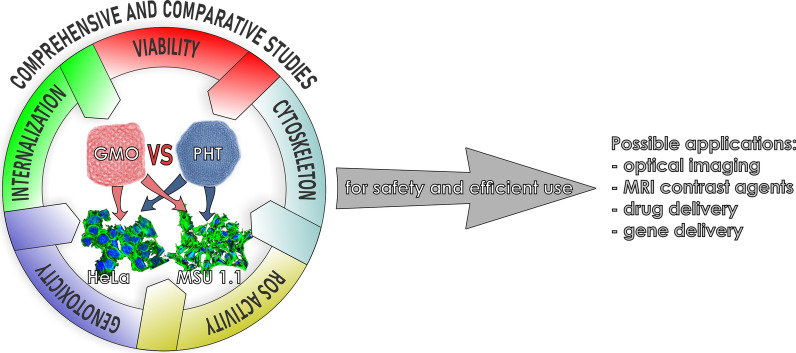

**Supplementary Information:**

The online version contains supplementary material available at 10.1186/s12951-021-00913-5.

## Background

Nanotechnology, which is being developed for several decades, emerges as a field of science and technology which is expected to influence a range of areas of human endeavor [1–5]. Currently nanomaterials and nanodevices more and more often find potential applications in everyday life, chiefly in the electronics applications [6–8], energy [9, 10] and environmental applications [11], food industry [12, 13], but also in medicine [14, 15] and pharmacy [16–18]. One of the main and most promising applications of nanomaterials in medicine and pharmacy is drug delivery. Although great milestones were already achieved, this field still faces numerous challenges. Therefore, novel and more advanced nanosystems are of great interest.

The nanomaterial designed for drug delivery ought to interlock plenty of features such as e.g., great drug accessible surface area, capability of solubilizing both hydrophilic and hydrophobic factors, sustained drug release, proper pharmacokinetics and inherent carrier nontoxicity for host’s cells [19–21]. Lipid liquid crystalline nanoparticles, a group of organic, self-assembled nanoparticles, meet the aforementioned requirements and therefore are considered as drug carrier. Depending on conditions such as temperature, pressure, lipid molecule geometry, lipid/water ratio or used stabilizer, different phases of LLCNPs can be distinguished such as: normal and inversed micelles, lamellar phase, normal and inversed hexagonal [22]. Very appealing for medical purposes are nanoparticles of inversed cubic phase, designated as cubosomes. Presence of two nonintersecting water channels separated with lipid bilayer enhances the surface area and creates both polar and nonpolar regions within LLCNPs. Owing to their capability of undergoing phase transitions, or through the functionalization, one can also easily control the drug release behavior [23, 24]. Moreover, most frequently used substrates for LLCNPs preparation i.e., glyceryl monooleate (GMO) and phytantriol (PHT) are FDA (Food and Drug Administration) approved and considered biocompatible. However, the impact of some structures in the nanodimension can have an unpredictable and completely opposite effect than their macroscale counterparts.

LLCNPs have already found many applications. Firstly, by the specific nature of LLCNPs, they have great potential to be used in theranostics as drug and imaging agent delivery systems. Nazaruk et al. [25] demonstrated the possible application of LLCNPs for delivery of doxorubicin in glioblastoma treatment. Tian et al. [26] presented folate-functionalized LLCNPs loaded with etoposide as a platform for imaging and therapy of cancer on the example of human breast carcinoma. Esposito et al. [27] fabricated cubosomes loaded with non-steroidal anti-inflammatory drug indomethacin for oral delivery. Murgia et al. [28] obtained GMO-based LLCNPs loaded with fluorescein and dansyl for a theranostics utilization. Similarly, a group of Nilsson [29] prepared phytantriol/oleic acid-based cubosomes and hexosomes functionalized with technetium-99 m as a contrast agent for SPECT/CT. Likewise, Alcaraz et al. [30] elaborated on cubosomes and metabolic labeling, which can be achieved by copper-free click chemistry. Further, Alcaraz et al. [31] reviewed the application of cubosomes as carriers for MRI contrast agents.

Elucidating the adverse effect of nanoparticles on living organisms is still one of the most important, emerging aspect for further development of nanomedicine. In order to do that special attention should be paid to understanding the mechanisms of nanoparticle-cell interaction and their repercussions from the point of view of morphology, biochemistry, and genetics. Nanoparticles can affect cells at various levels. The communication between nanoparticles and cells begins at the cellular membrane, where, depending on size, shape and surface chemistry nanoparticles can be internalized or not into the intracellular environment [32]. Inside the cell the fate of nanoparticle is still not clearly defined. The gene expression can be also altered indirectly, for example by interaction and misbalancing redox cellular system [33]. Changes in the redox system can further alter cellular signaling pathways, the structure of the genome, organelles such as mitochondria and nucleus or constitutional structures such as cytoskeleton [34]. All these events can eventually induce apoptosis and lead to cell death. Therefore, it is crucial to investigate how specific nanoparticles interact with given cells, and hence one can design an effective treatment strategy for a given type of cell. So, as a result, one can design effective treatment strategy.

Further development of the drug and/or imaging agent delivery system based on LLCNPs strongly relies on their chemical and structural resemblance to the cell membrane, owing to which these LLCNPs may reflect cell membrane properties and functions. Therefore, the comprehensive studies of their cytotoxicity profiles but also their broadly understood interaction with cells are of high importance. Consequently, the LLCNPs-based drug carriers with the required properties can be designed and fabricated for the specific drug and imaging agent delivery. Therefore, the purpose of the study was to provide such comprehensive cytotoxicity and genotoxicity assessment of LLCNPs prepared from distinct types of lipids, namely GMO and PHT towards cervical cancer cells (HeLa) and human fibroblast cells (MSU 1.1) for the comparison. These studies were followed with the examination of LLCNPs internalization pathway, evaluation of cytotoxicity based on cells metabolic activity, investigation of cytoskeleton integrity, measuring intracellular ROS generation and assessment of gene expression profiles upon treatment with the lipid liquid crystalline nanoparticles.

## Results

### Formulation and physicochemical description

Prepared LLCNPs colloidal dispersion appeared homogeneous milky white for both GMO and PHT-based formulations. The 20 w/w% formulation exhibits a highly viscous character.

**Fig. 1:**
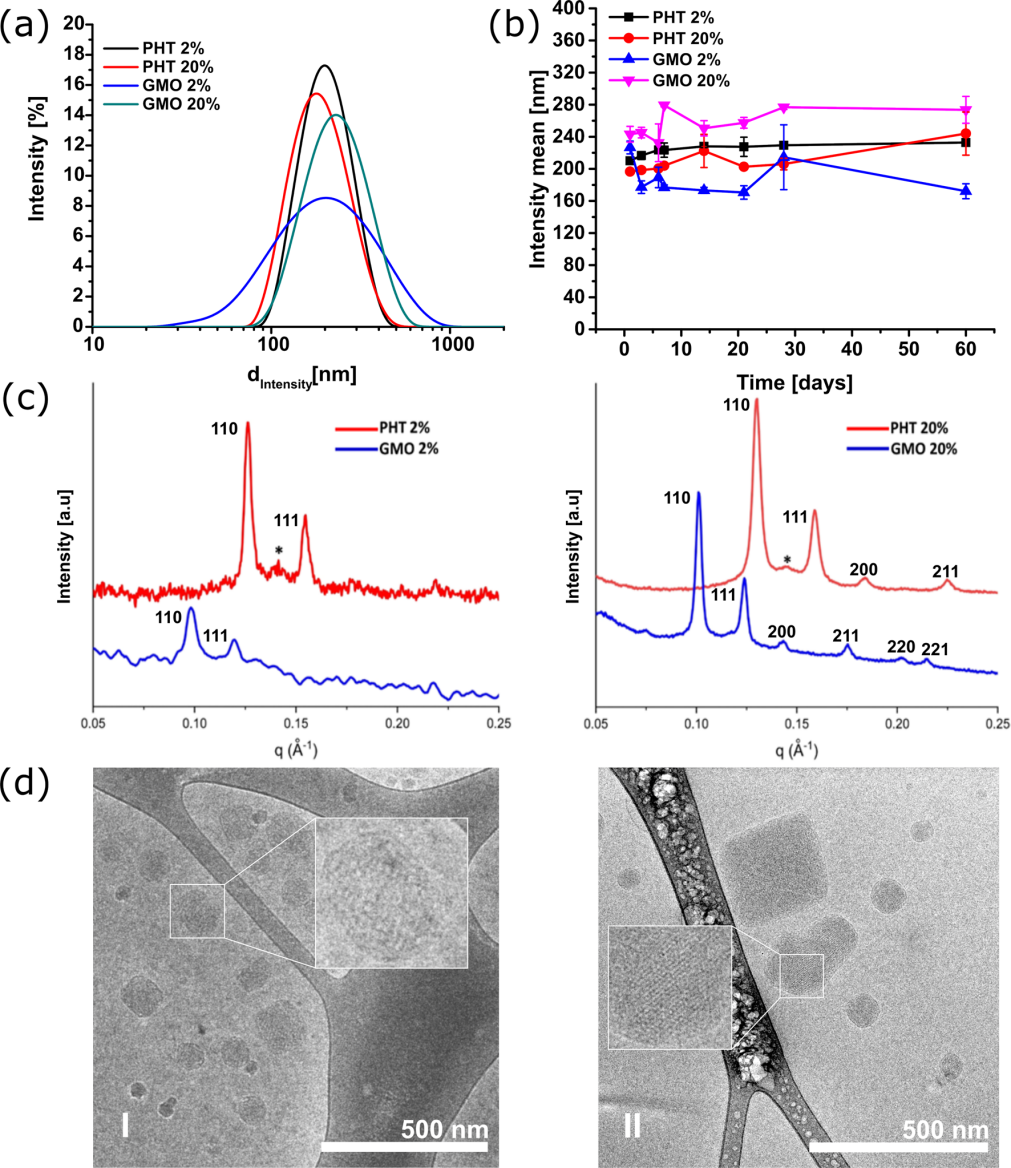
Physicochemical characterization of fabricated LLCNPs. **a** Dynamic Light Scattering (DLS) intensity weighted particle size distribution curves of PHT and GMO LLCNPs, **b** Intensity weighted particle diameter of LLCNPs measured over a period of 60 days, **c** Scattering curves for PHT and GMO LLCNPs at 20 °C—the asterisk in SAXS plots indicates peak characteristic for liposomes. **d** Cryo-TEM images of I—GMO 2% and II—PHT 2% LLCNPs

Particle size distribution measurements performed after one day since the preparation of LLCNPs (Fig. 1a) show that the smallest particles were obtained for PHT (196 nm for PHT 2%) and the biggest for GMO formulations (243.1 for GMO 20%). The latter also exhibited the broad size distribution, which is reflected in the highest polydispersity index (PdI) (0.353 and 0.209 for GMO 2% and GMO 20%, respectively) (Table 1) in comparison to other formulations. Measured PdI values are in general lower than 0.5, which indicates that prepared dispersions have monomodal particle size distribution. Prepared LLCNPs dispersions are in general negatively charged, of fair colloidal stability. The obtained particles size and ζ potential values over the 60 days (Fig. 1b) show that prepared LLCNPs maintain their size and thus colloidal stability.

**Table 1:** DLS/ζ potential measurements for different LLCNPs formulations after equilibration at RT for 24 h

Sample	d_(intensity)_/SD [nm]	PdI/SD	Ζ potential/SD [mV]
PHT 2%	210.1/1.3	0.102/0.031	−23.6/0.1
PHT 20%	196.6/1.0	0.102/0.012	−24.3/1.0
GMO 2%	226.7/8.0	0.353/0.003	−15.2/0.5
GMO 20%	243.1/9.9	0.209/0.021	−22.0/1.2

*d*_*(intensity)*_ intensity-weighted diameter

The results of cryo-TEM studies confirmed that both of studied system revealed cubic structure, however besides very well-defined bicontinuous forms of nanoparticles there are populations of other common forms of self-assembled systems like liposomes or less organized, disordered, not fully formed cubic forms (Table 1). The obtained data for GMO are in accordance with previous studies reported by Flak et al. [35] Also PHT LLCNPs are comparable in terms of morphology to system obtained by other groups with top-down fabrication methods [36, 37]. The estimated size of LLCNPs based on cryo-TEM measurements is ranging from tens to hundreds of nanometres and is fairly similar to reported DLS data.

As the critical step of the physicochemical analysis, SAXS measurements were performed to confirm specific ordering of lipids within nanometric particles. The results for two different concentrations for both kind of lipids carried out at 20 °C are shown in the Fig. 1b. In the presented *q* region four peaks for PHT and six for GMO were observed and resolved as characteristic for *Pn3m* structure [38, 39]. The calculated from SAXS data mean lattice parameters are presented in the Table 2. In the profile of scattering curves (Fig. 1b) of PHT 2 and 20% there are additional small peaks (marked with an asterisk) visible at *q* value of about 0.15, which could be attributed to the existence of liposomes in the colloidal dispersion [40]. Based on the ratio of peak intensities attributed to *Pn3m* like structures to liposome’s one, it can be concluded that in the case of PHT 2% the number of lipid nanoparticles in form of liposomes might be higher than in dispersion of PHT 20%. It is also worth to underline that the mean lattice parameter is slightly smaller for samples at 20% concentration of lipids, what could be attributed to lower efficiency of lipid hydration in more concentrated dispersion and thus less swollen, however not necessarily smaller, lipid structures with decreased lattice constants.

**Table 2:** The mean lattice parameters for LLCNPs calculated from scattering curves

Sample	PHT	GMO
Concentration		
2%	7.03 ± 0.02 nm	9.03 ± 0.04 nm
20%	6.83 ± 0.01 nm	8.76 ± 0.02 nm

### Cytotoxicity

**Fig. 2:**
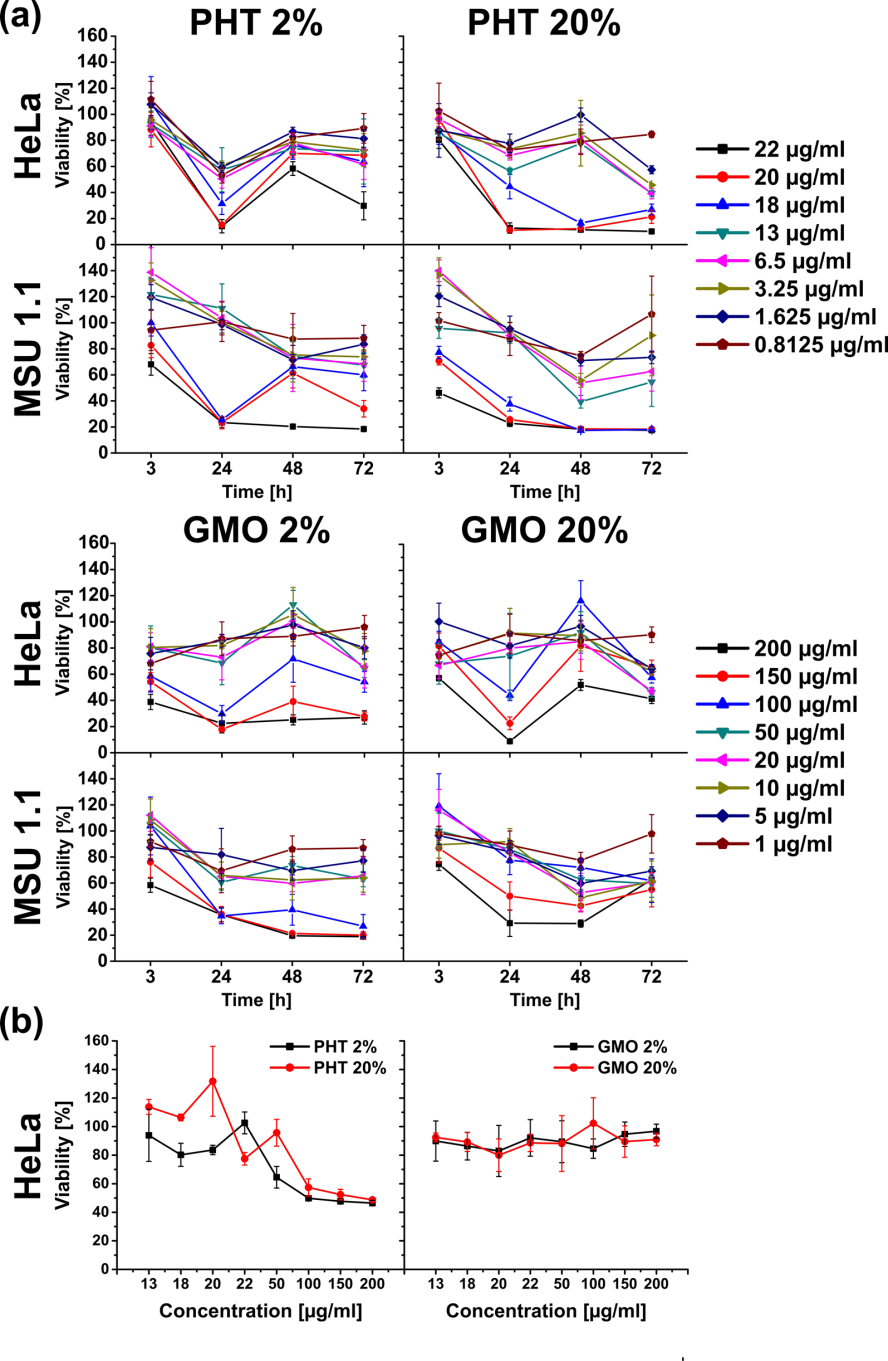
Viability studies with WST-1 assay. **a** HeLa and MSU 1.1 cells monolayers, **b** HeLa spheroids upon incubation with LLCNPs. The statistical analysis is included in Additional file 1: Table S1

The time-dependent viability was evaluated using WST-1 assay and results were expressed as a ratio of the incubated cells absorbance to the absorbance of control. Results differ between PHT and GMO LLCNPs formulations, where in most cases the drop in viability was observed at 18 µg/ml and 100 µg/ml LLCNPs, respectively (Fig. 2a). After 3 h of incubation, viability of HeLa cell line does not differ significantly in whole range of concentration just for PHT 2% vs PHT 20%. In case of other samples the most evident differences in viability after 3 h were found for GMO 2% vs GMO 20% incubated with Hela cells, as well as PHT2% and 20% in MSU1.1 cells at the highest concentrations (100–200 µg/ml). Concerning 24 h treatment, a great decrease in viability is observed at the highest concentrations, i.e., 100–200 µg/ml for GMO-based LLCNPs and 18–22 µg/ml for PHT-based LLCNPs. Surprisingly, in the case of 48 h and 72 h incubation, the recovery of the cells is observed and the viability reached again about 60% of control (Fig. 2a). The possible reason of slight differences in cytotoxicity especially between PHT 2% and 20% formulations, could be different cubosomes to small, but existing, fraction of liposomes ratio in colloidal dispersion what was found from morphological and structural characterization (Fig. 1c) [41].

Concerning spheroids, the results are comprehensively different (Fig. 2b). GMO-based LLCNPs exhibit no significant impact on HeLa spheroids, remaining viability around 90% at all concentrations, i.e., up to 200 µg/ml. In turn PHT-based LLCNPs seem to be more toxic for HeLa spheroids than GMO-based ones. At lower concentrations (up to 20 µg/ml) the viability in spheroids for both PHT 2% and PHT 20% remains at similar level as for GMO-based LLCNPs. At concentration 50 µg/ml of PHT 2%, the viability of cells in spheroids drops down reaching 50% at 100 µg/ml. Meanwhile cell treated with PHT 20% formulations exhibit no differences in viability at 50 µg/ml in comparison to GMO-based LLCNPs, however at 100 µg/ml have similar effect to PHT 2%, decreasing the viability to 50%.

Taking into account the dose-dependent cytotoxicity screen, the concentrations of 8 µg/ml and 16 µg/ml were selected for further studies concerning ROS generation, cytoskeleton organization and genotoxicity of PHT-based LLCNPs; whereas in case of GMO-based LLCNPs the higher concentrations from 25 µg/ml up to 100 µg/ml were used.

### Reactive oxygen species activity

The ROS cellular activity was evaluated by means of the CellRox® Green assay, mainly used to evaluate the superoxide (O_2_^−^) and/or hydroxyl radical (^•^OH) in live cells and the DCFH-DA assay, which is commonly used to measure the level of hydroxyl (^•^OH), peroxyl (ROO^•^) and other ROS activity within the cell.

**Fig. 3:**
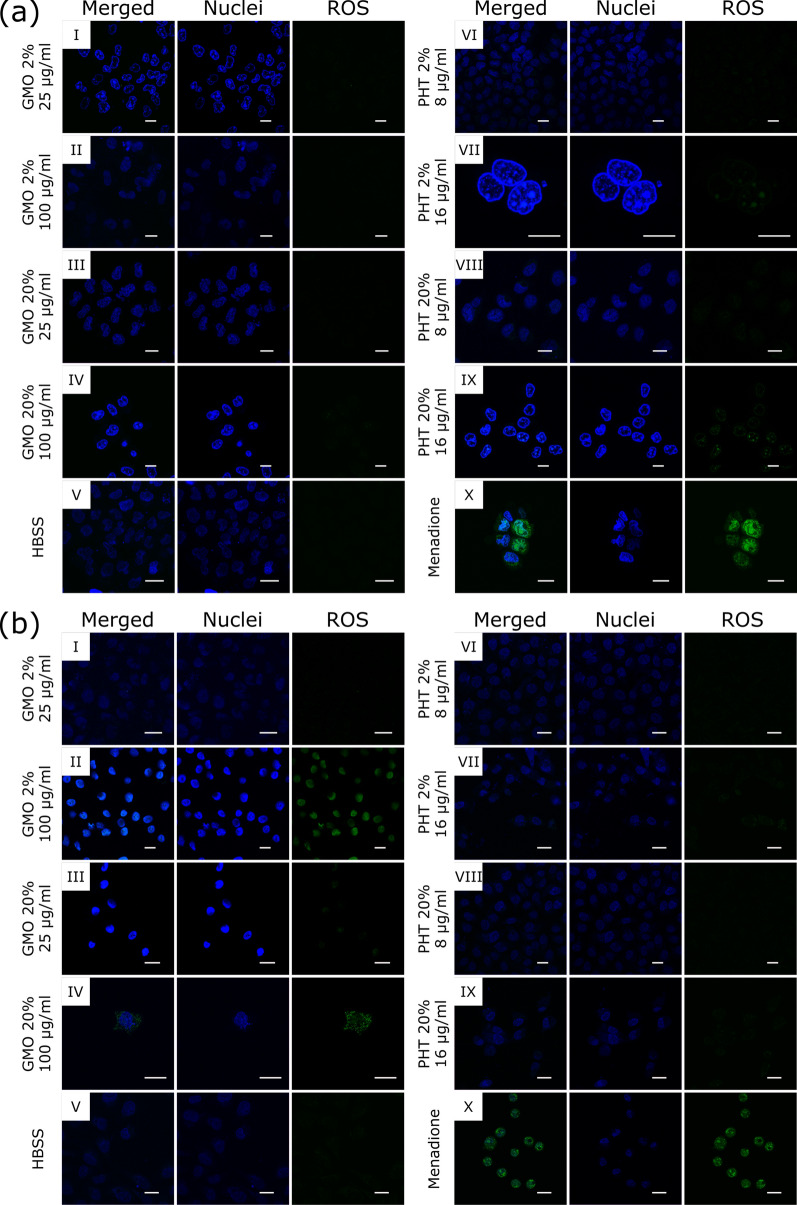
Visualization of reactive oxygen species generation in **a** HeLa and **b** MSU 1.1 cells upon treatment with LLCNPs. Scale bar: 20 µm

Results of the CellRox® Green assay shows that in HeLa cells incubated with 2% and 20% GMO-based LLCNPs, the signal at both high (100 µg/ml) (Fig. 3a II, IV) and low (25 µg/ml) (Fig. 3a I, III) concentrations is comparable to the negative control (Fig. 3a X). When the cells were treated with PHT-based LLCNPs similar results are observed during the treatment with lower (8 µg/ml) concentration of both 2% and 20% PHT-based LLCNPs (Fig. 3a VI, VIII). The ROS signal is however observed in Hela cells incubated with higher concentration (16 µg/ml) of both 2% and 20% PHT-based formulations (Fig. 3a VII, IX). Nevertheless, the signal is weaker than in a positive control.

When MSU 1.1 cells were incubated with GMO-based LLCNPs, only at low concentration (25 µg/ml) of GMO 2% no ROS-related signal is observed (Fig. 3 I). The treatment with high concentration (100 µg/ml) of GMO 2% (Fig. 3b II) generates ROS signal similar to a positive control. Finally, the treatment with both lower and higher concentrations of GMO 20% formulations (Fig. 3b III, IV) results in an increased signal in comparison to positive control, particularly in case of higher nanoparticles concentration.

In turn the treatment of MSU 1.1 cells with PHT 2% and 20% LLCNPs at low concentration (8 µg/ml) (Fig. 3b VI, VIII) results in no ROS-related signal, similarly as in a negative control. The signal, however weak, was registered only for cells treated with higher concentration (16 µg/ml) of both PHT 2% and PHT 20% formulations (Fig. 3b VII, IX).

Subsequently, the DCFH-DA assay was conducted. In case of HeLa cells, it is worth noticing at first, that for negative (HBSS) and positive (H_2_O_2_) controls the ROS levels are not significantly different (p > 0.05), but still with slightly higher relative ROS-related fluorescence intensity for positive control. This is not the case for MSU 1.1 cells, where the difference between negative and positive control is significant (p ≤ 0.01). The presence of ROS in negative control of HeLa cells may be an effect of glucose deprivation-induced oxidative stress, which is a metabolic effect characteristic of human tumor cells [42].

**Fig. 4:**
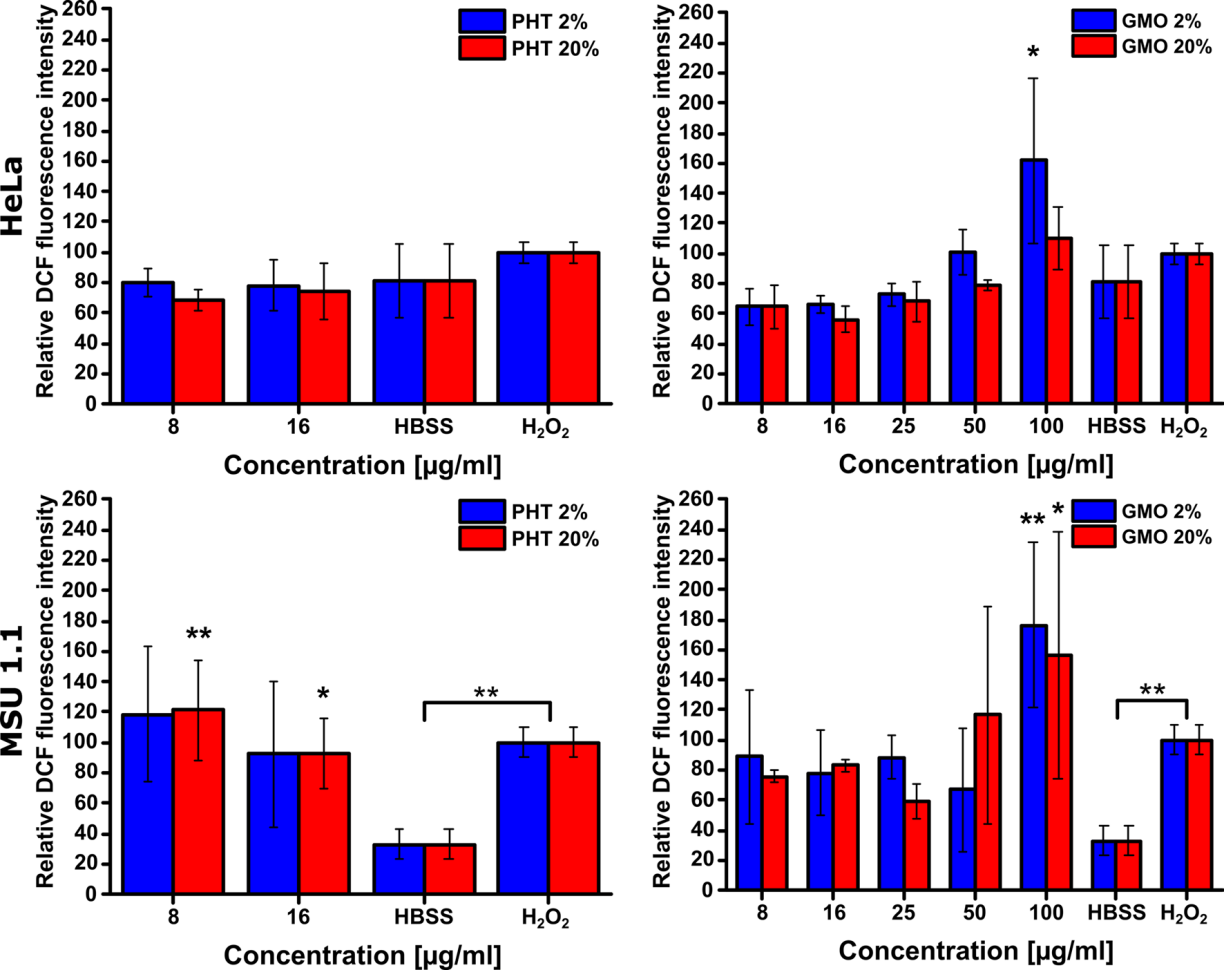
Quantitative evaluation of reactive oxygen species (ROS) generation in HeLa and MSU 1.1 cells using DCFH-DA assay. Results presented as a relative DCF fluorescence intensity of ROS generation as a function of LLCNPs concentration, compared to HBSS and H_2_O_2_ controls, respectively negative and positive. Asterisks denotes the statistical significant difference compared with controls *—p ≤ 0.05, **—p ≤ 0.01

The level of ROS-related fluorescence intensity in HeLa cells incubated with PHT 2% and 20% LLCNPs at both lower (8 µg/ml) and higher (16 µg/ml) concentration is at comparable level to a negative control (p value 0. ≤ 05 for PHT 20% 8 µg/ml, p value > 0.05 for PHT 2%, PHT 20% 16 µg/ml). Likewise, in HeLa cells treated with GMO 2% and 20% LLCNPs at lower concentrations (8 µg/ml to 25 µg/ml), the signal is similar to a negative control. The increase of ROS-related intensity is however observed for HeLa cells treated with higher concentration of 50 µg/ml reaching the similar level of a positive control. Finally, the ROS signal reaches the highest value for cells treated with 100 µg/ml of GMO LLCNPs (particularly GMO 2%) surpassing even the intensity level of a positive control (Fig. 4).

In case of MSU 1.1 cells, similar behavior is observed upon treatment with both types and the same concentrations of LLCNPs, however prepared formulations at lower concentrations (8 µg/ml for PHT-based, 8 µg/ml and 16 µg/ml for GMO-based LLCNPs) seem to induce slightly higher levels of ROS in comparison to the same experiments with HeLa cells. These DCFH-DA assay results are therefore in agreement with the microscopic analysis of ROS-generation using the CellRox® Green assay described above.

### Cytoskeleton integrity

**Fig. 5:**
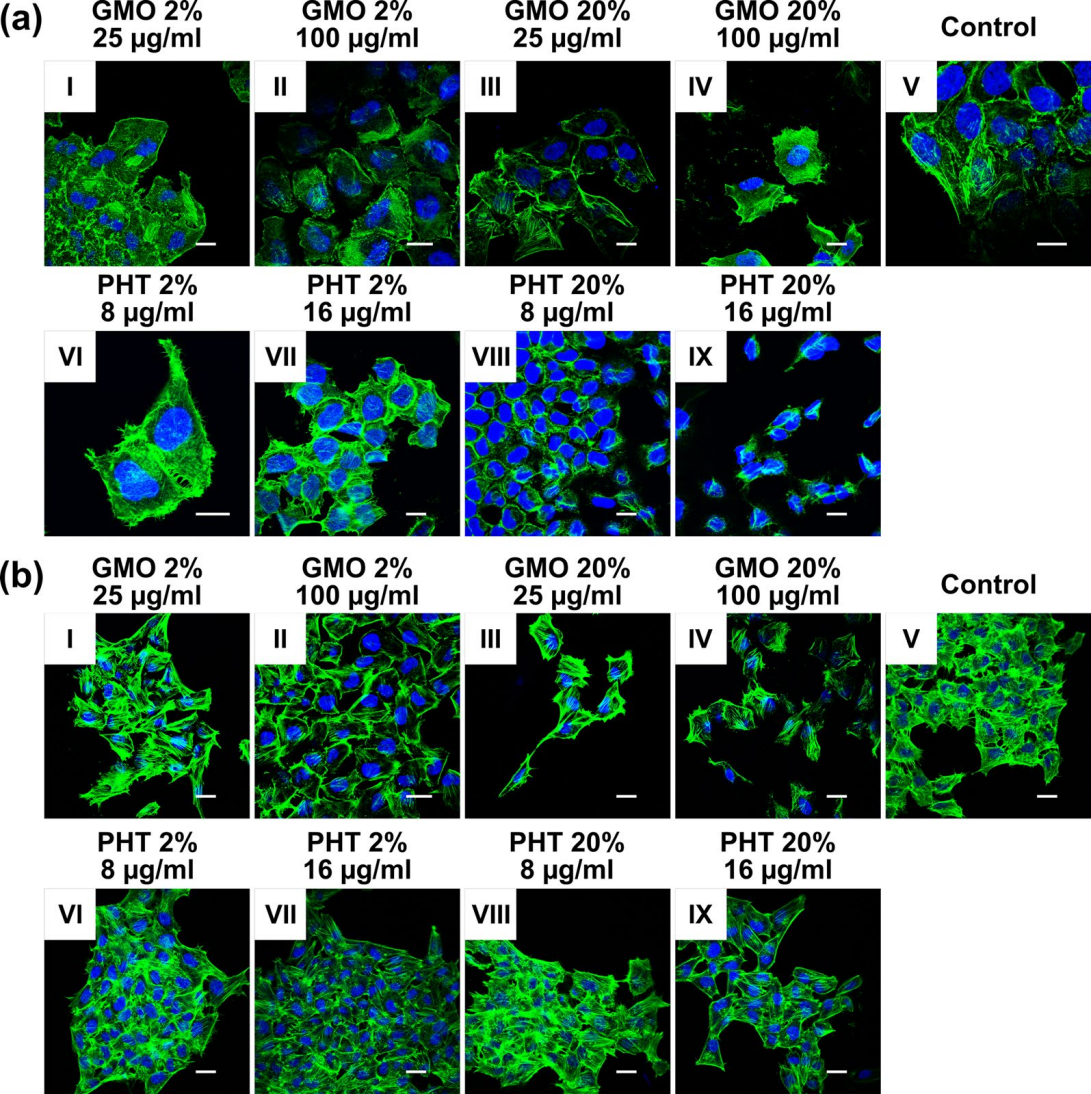
Integrity of cytoskeleton in **a** HeLa and **b** MSU 1.1 cells upon treatment with LLCNPs. Scale bar: 20 µm

Cytoskeleton integrity studies show that HeLa cells emerge as more vulnerable to cytoskeleton degradation upon incubation with LLCNPs. The GMO compositions, at both concentrations of 100 µg/ml and 25 µg/ml (Fig. 5a I–IV), influence the cytoskeleton’s integrity, however at higher concentration the cytoskeleton morphology is visibly more distorted. PHT-based LLCNPs appear to be more harmful than GMO-based ones, especially regarding PHT 20% formulation, which completely degraded the cytoskeleton at both higher (16 µg/ml) and lower (8 µg/ml) concentrations (Fig. 5a VIII, IX).

In case of MSU 1.1, treatment with both PHT and GMO formulations appear to be less harmful than for HeLa cells, however changes in cytoskeleton morphology are still visible. For PHT 2% and PHT 20%, at lower concentration, cytoskeleton structure is comparable to the control (Fig. 5b VI, VIII), while at 16 µg/ml the disruption in F-actin filaments is noticed (Fig. 5b VII, IX). Regarding GMO-based LLCNPs, a similar effect is observed as described above, however at both concentrations the cytoskeleton morphology differs from the control (Fig. 5b I–IV).

### Genotoxicity

In presented studies, the expression of *GADD45A1*, *DHFR*, *CDK1*, *ACTB* genes was investigated. Genes were selected based on their role in DNA damage or significant metabolic processes. Expression profiles of selected genes in normal human cells (MSU 1.1) as well as in cancer cells (HeLa) were determined by real-time PCR after exposure to increasing concentration of 2% and 20% PHT–based and GMO-based LLCNPs. The gene expression changes were assessed with GAPDH as the normalizer reference gene.

**Fig. 6:**
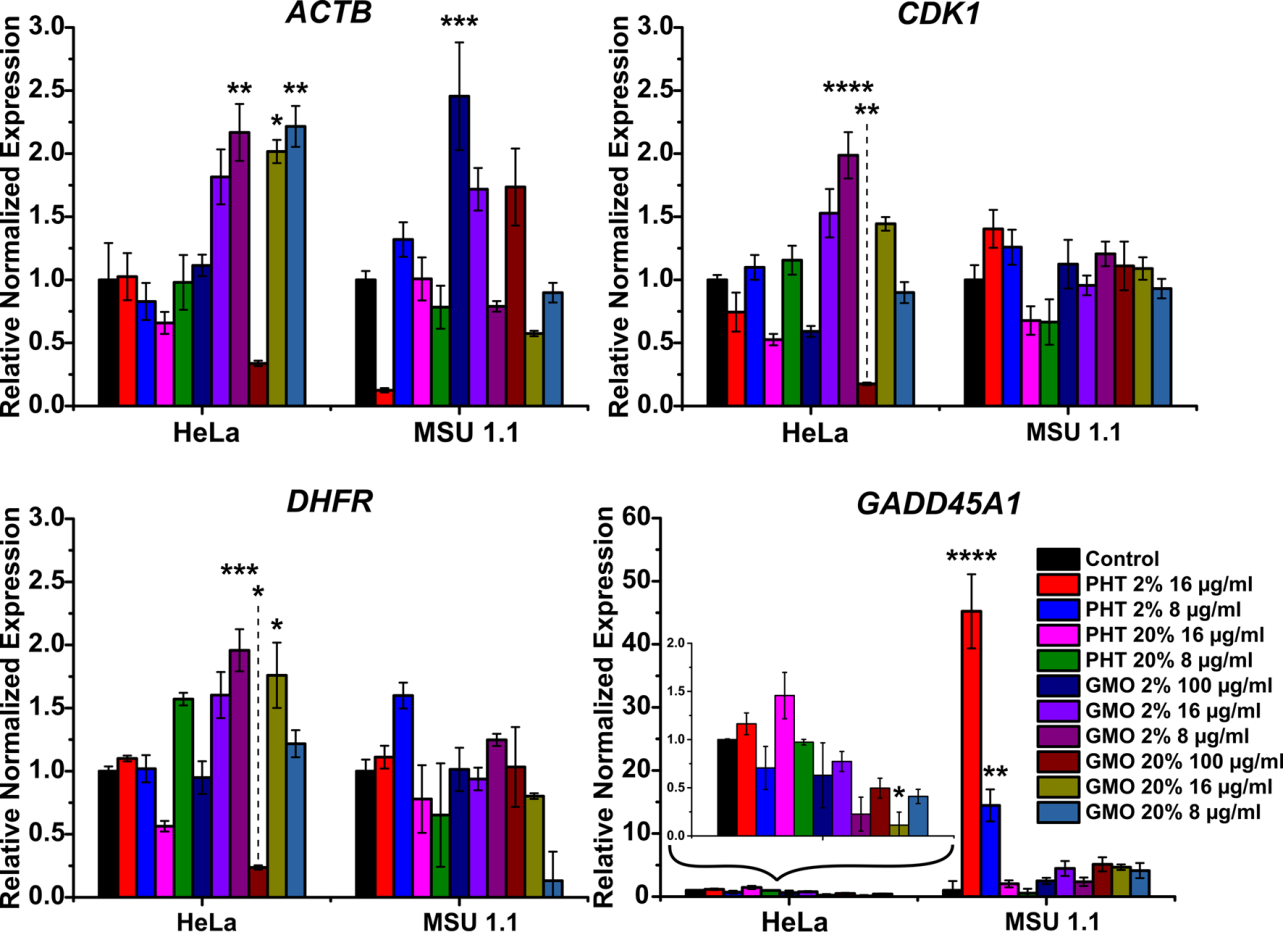
Relative normalized expression of ACTB, CDK1, DHFR, GADD45A1 genes in HeLa and MSU 1.1 cells upon incubation with different LLCNPs formulations. Asterisks denotes the statistical significant difference compared with controls *—p ≤ 0.05, **—p ≤ 0.01, ***—p ≤ 0.001, ****—p ≤ 0.0001

As it was performed in previous experiments specific concentrations’ range for these two samples was selected. The presence of all gene transcripts was revealed in all examined samples; however, their expression patterns differ (Fig. 6).

The analysis of *DHFR* gene expression in MSU 1.1 cell line shows generally similar expression level for cell treated with all formulations, except the GMO 20% treated cells, which exhibits a downregulated expression at its lowest concentration. The analysis of *DHFR* gene expression in HeLa cells indicates downregulation after PHT 20%- and GMO 20%-based LLCNPs treatment at the highest concentration i.e. 16 µg/ml and 100 µg/ml, respectively. The upregulation can be observed only for GMO-based LLCNPs, including GMO 2% at 8 µg/ml and GMO 20% at 16 and 25 µg/ml concentration. Interestingly, for GMO 2% after gene upregulation at the lowest concentration, one could observe decreasing in a concentration-dependent manner to the level of control for the rest of the samples. While for the GMO 20%, gene expression level increased together with the concentration till 25 μg/ml and after that decreased to the lowest level at 100 µg/ml, where the *DHFR* gene is downregulated.

The level of *GADD45A1* gene expression in MSU 1.1 cells increased very strongly in PHT 2%-based samples compared to others, where it remains at the level of control. On the other hand, in HeLa cells the gene expression after PHT-based samples treatment remains at the control level, while the GMO-based exhibit lower level, except GMO 2% at 25 µg/ml concentration, where slight increase could be observed.

In case of *CDK1* gene, the expression level in MSU 1.1 cells was generally unchanged regarding untreated control for both LLCNPs. However, in HeLa cells the upregulation of *CDK1* gene is visible for cells treated with GM0 2% at the lowest concentration, whereas for cells treated with GMO 20% the downregulation of the gene is observed at the treatment with the highest concentration.

The analysis of *ACTB* gene showed that its expression level increased in a non-concentration dependent manner in both cell lines. In MSU1.1 this gene was downregulated at the high concentration of PHT 2% sample and interestingly upregulated at the highest concentration of GMO 2%. In HeLa cells beta-actin expression in PHT-based samples remains at the similar level of control, whereas GMO-based were upregulated, except the highest concentration of both GMO samples.

### Internalization pathways

**Fig. 7:**
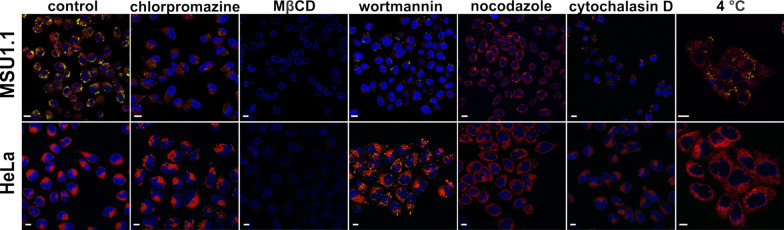
CLSM images of living HeLa and MSU 1.1 cells incubated with stained LLCNPs and with selected transport inhibitors. Red and green (lipid droplets) signals are LLCNPs, blue – nuclei. Scale bar: 10 μm

Taking into account that, GMO 2% LLCNPs sample was considered the most attractive for future research, it was decided to study in detail the cellular internalization mechanism on two cell lines MSU1.1 and HeLa. For these studies Nile Red stained GMO 2% at a concentration 25 µg/ml was used. As it is shown at Fig. 7 for control sample both cell lines are efficiently internalized after one-hour incubation with LLCNPS, what is observed as a red signal located in the cytoplasm, close to the nuclear membrane. Additional green spots, which are mainly visible in case of the MSU1.1 cell line, are characteristic for lipid droplets staining by Nile Red [43].

To determine whether LLCNPs uptake was by energy-dependent or -independent mechanism, cell cultures were incubated at 4 °C to inhibit active processes by inactivation of the temperature-fragile enzymes. Interestingly, exposure to GMO 2% at lowered temperature did not result in an inhibition of endocytosis in both cell types. Then, by using suitable inhibitors, different types of endocytosis were investigated. The uptake inhibition by methyl-β-cyclodextrin, an inhibitor of lipid raft formation, indicates the role of cholesterol in LLCNPs internalization. As can be seen in Fig. 7. the depletion of cellular cholesterol resulted in 100% inhibition of GMO 2% uptake for both cell types. Moreover, MSU1.1 cells treated with cytochalasin D showed that it affects nanoparticles internalization. Cytochalasin D is responsible for F-actin depolymerization, one of the two major constituents of the cytoskeleton structure, which in turn plays an important role in multiple cellular events, including endocytosis and trafficking of endocytosis vesicles. In the Fig. 7, the red signals coming from Nile Red were decreased in MSU1.1 cells, but slightly also in HeLa cells relative to control. A similar effect was observed also for wortmannin, which is an inhibitor that blocks the action of phosphoinositide 3-kinase, a key regulator in macropinocytosis. The fluorescence signal in MSU1.1 is decreased in comparison to control. In the case of cells treated with chlorpromazine and nocodazole, the red signal in both of them remained unchanged, which means that these two inhibitors did not affect nanoparticles internalization.

Additionally, the internalization was performed on 3D tumor spheroid. Nile Red stained GMO 2% LLCNPs were also incubated with HeLa cellular spheroids and confocal imaging was performed in order to analyze the internalization efficiency. The fluorescent signals of Nile Red were observed dispersed evenly throughout tumor cells (Additional file 1: Fig. S1), indicating a high permeation.

## Discussion

This study demonstrates the comprehensive investigation of GMO- and PHT-based LLCNPs, which were so far commonly recognized as efficient drug delivery vehicles into cells, due to their increased chemical and physical stability of such formulations comparing to the liposomes [44]. However, numerous previous studies provide not sufficient and not consistent information regarding their cytotoxicity, genotoxicity and related biological behavior. Therefore, the benefit of LLCNPs in nanomedicine as drug and /or imaging agent delivery is strongly inhibited.

Prepared here typical LLCNPs are three component systems: water, lipid (commonly used GMO and PHT), and also widely used polymer surfactant Pluronic F-127. In hydrated bulk phase both lipids exhibit long range cubic symmetry [45, 46]. The applied here the top-down synthetic approach, based on the high energy sonication method of hydrated bulk lipid phase and the addition of polymer surfactant, yielded nanodimensional particles, which were found to exhibit mainly bicontinuous cubic structure of *Pn3m* symmetry. As a result, GMO and PHT LLCNPs as dispersed phase formulations showed good colloidal stability and maintained the size and the particle ζ potential over the time, which is important from an applicability point of view.

Both GMO and PHT have specific advantages and some essential disadvantages as structure-forming lipids for LLCNPs. GMO is classified as generally safe material, FDA approved and commonly used in the food industry. PHT is in turn considered more harmful than GMO, causing membrane disruption [37], however, at lower concentrations it is used as an additive in cosmetics [38]. Moreover, due to the absence of double bond in chemical structure, PHT-based LLCNPs are more suitable than GMO ones [44], and this was also shown in our studies.

Cytotoxicity of GMO- and PHT-based LLCNPs with 2 w/w% and 20 w/w% lipid content were investigated to find out whether it is possible, that even subtle morphological and structural changes within LLCNPs of different formulation can influence their biocompatibility. According to WST-1 assay, which relies on the metabolic activity of cells, after 3 h incubation, first signs of cytotoxicity were observed. For most cases, the viability of cells decreased with increasing concentration of nanoparticles, except for HeLa cells treated with PHT- based LLCNPs, both 2% and 20% formulations. The greatest drop in viability occurred after 24 h incubation at a concentration above 100 µg/ml for GMO and 13 µg/ml for PHT with little deviations. However, in the case of 48 h, regarding 2% PHT formula for both cell lines, and both GMO formulas for the HeLa cell line, cells commenced to recover. Concerning impact of PHT based LLCNPs on cells, they appear to be more toxic than GMO ones. Greater cytotoxicity of PHT LLCNPs may be thus due to the disruption of the cell membrane and not due to the surfactant [37]. The restoration of cells after 48 h and 72 h incubation, even with highest LLCNPs concentrations can be explained as a result of progressive metabolism of nanoparticles. As a result, cells which managed to survive, restored the whole culture, apart from the concentrations, at which damage was irreversible. Obtained data are in accordance with previously reported by Hinton et al. [37] where cytotoxicity of GMO and PHT based LLCNPs on CHO and A549 cells was investigated.

Further the effect of GMO and PHT-based LLCNPs on cells was reflected in the ROS generation ability experiments. Results show that detected generated ROS levels upon exposure to prepared LLCNPs are in general dose-dependent and are increasing with rising concentration for all formulations. However, the generated ROS level in HeLa cells remained lower or similar value than for the negative control, apart from both GMO formulations at higher doses (50 and 100 µg/ml). The stronger ROS generation ability of prepared LLCNPs was observed in MSU 1.1, however, the effects still do not exceed significantly the positive control, as above with the exception of both GMO formulations at higher doses. Falchi et al. [43] has already presented that the cellular uptake of LLCNPs (cubosomes) leads to the mitochondrial hyperpolarization and hence mitochondrial ROS generation next to the modification of the cell lipid profile and lipid droplet accumulation. This may further have an effect on subcellular organelles, such as nucleus and mitochondria, by damaging DNA, and their possible adverse effects on cell functions [47]. However, the presented study shows that prepared GMO and PHT-based formulations do not exhibit significant ROS generation ability within the applied concentration range, and thus should not be considered as the significant source of cytotoxic effects.

Moreover, it was proved that LLCNPs have an impact on the cytoskeleton integrity. Cytoskeleton is a system of proteins including actin filaments, microtubules and intermediate filaments, responsible for maintaining the cell structure, the intracellular transport, signal transduction, motoric features, organelles localization, internalization and adhesion [48–50]. While cytoskeleton plays function in so many processes, evaluating the adverse effect of nanoparticles on it appears to be crucial in the cytotoxicity research [51].

Concerning PHT formulation for both 2% and 20%, no adverse effect on MSU 1.1 cells cytoskeleton integrity was observed. Both at higher (15 µg/ml) and lower concentration (7.5 µg/ml) F–actin filaments preserve similar structure to control. However, in HeLa cells, at higher concentration, the disruption of cytoskeleton was noticeable. The analogous effect occurred in GMO-based formulation. While in MSU 1.1 cells no harmful effect was detected, in HeLa cells, at the highest concentration of LLCNPs (100 µg/ml) cytoskeleton was disrupted. These results suggest that HeLa cells are more prone to both GMO- and PHT- LLCNPs, however no such differences were observed after WST-1 assay. According to Scoville et al. [52], hydrophobic loop of actin is connected with filament stability. Hence, while Pluronic F-127 has a highly hydrophobic region, it may physically block access to the opposite strand and thus interfere with stability. Ispanixtlahuatl-Meráz et al. [51] highlighted also other ways of interaction of organic nanoparticles with cytoskeleton, such as epigenetic changes in cytoskeleton associated genes or accretion of autophagosomes. At this point however, it is difficult to decide which mechanism occurs and why MSU 1.1 cells are not susceptible to the harmful effect. Further, the cytoskeleton integrity may be also associated with cell adhesion properties [53, 54].

In order to evaluate the impact of obtained LLCNPs on cells at the molecular level and thus to better understand the underlying molecular mechanism of nanotoxicity, the RT qPCR was performed. For genotoxicity analysis four genes involved in DNA damage or important metabolic processes were chosen and their expression were further analyzed in normal and cancer cell lines after LLCNPs treatment.

*GADD45A1* (Growth Arrest And DNA Damage Inducible Alpha 1), which is known to be highly expressed in response to presence of DNA-damaging factors as well as in stressful growth-arrest conditions and during the apoptosis [55]. It was previously reported that exposure to nanoparticles such as chitosan NPs [56] and CuO NPs [57] led to upregulation of *GADD45A1* in cells. Here, the expression level was increased significantly only in case of PHT2%-based LLCNPs, indicating that this pathway is affected in case of these PHT-based samples at selected concentration range.

Cell cycle machinery is controlled by cyclin-dependent kinase (CDK), cyclins and CDK inhibitory proteins. The changes in CDK gene expression led to eukaryotic cells division disorders and apoptosis. This gene was often upregulated in other studies, indicating that nanoparticles could affect cell proliferation and division for example after exposure to CuO NPs [57] or AgNPs [58]. However, our results showed that the gene expression of *CDK1* is generally unaffected, hence it the LLCNPs does not lead to the cells apoptosis.

*DHFR* encodes Dihydrofolate Reductase protein, which is involved in folate metabolism [59] and in de novo synthesis of glycine and purine aminoacids, functioning of mitochondria and, according to GeneCards database (GC05M080626), has significant role in cell growth and proliferation and thus is the crucial gene in cell development. As investigated, LLCNPs did not affected *DHFR* gene expression except two minor cases of downregulation in MSU 1.1 cells incubated with GMO 20% at concentration 8 µg/ml and HeLa cells incubated with GMO 20% at concentration 100 µg/ml.

The actin family consists of highly conservative proteins, abundant in all eukaryotic cells. Globular actin polymerizes to produce filaments that form cross-linked networks in the cytoplasm of cells. Moreover, cytoskeleton plays key functions, such as cell motility, contraction, maintenance of cell shape, signal transduction and cell adhesion. In addition to their role in the cytoplasmic cytoskeleton also localize in the nucleus, and regulate gene transcription and motility and are involved in the repair of damaged DNA [60]. Taking into account that *ACTB*, the cytoplasmic actin isoforms β are ubiquitously expressed and essential for cell functioning, this gene is usually used as a reference in gene expression studies [61]. However, as it was presented in the cytoskeleton studies the LLCNPs affect the cell cytoskeleton, especially at higher concentration, suggesting changes in actin genes expression after nanoparticles exposure. Also, in our previous work [35] the changes in actin gene expression profile under stress condition related with cubosomes treatment was observed. However, in contrary to current results, the gene expression was downregulated.

The internalization studies performed on the GMO-based LLCNPs on normal and cancer cell lines indicated efficient uptake and localized the nanoparticles in the cytoplasm, close to perinuclear membrane. This intracellular localization was observed also in other research concerning LLCNPs [62, 63]. Moreover, in normal human fibroblasts the hydrophobic lipids were visible, indicating progressive accumulation in lipid droplets [43]. It is consistent what was reported by Faria et al. [62] that GMO-based cubosomes use lipid droplet compartments to transport fatty acids until the mitochondria, where they are metabolized afterwards. The additional analysis of GMO-based LLCNPs uptake mechanism indicated, that they were internalized mainly by the energy-independent and cholesterol-dependent manner. This type of uptake together with time-dependent internalization of LLCNPs was previously observed by Deshpande et al. [64] in HeLa cells as well as murine fibroblast cell line (NIH3T) and a breast cancer cell line (MDA-MB231). This kind of LLCNPs—cell membrane interaction could be related to the membrane fusion as it was described by Dyett et al. [65]. However, other uptake mechanisms’ types could be also considered for such types of nanoparticles. As an example, the endocytosis–independent mechanism of internalization of hexosomes was previously proposed [66]. In this case, authors indicated the crucial role of interaction between the membrane-lipids and nanoparticles, membrane destabilization and direct nanoparticles translocation. Additionally, for normal human fibroblasts the role of macropinocytosis in LLCNPs internalization was observed. This kind of internalization was found to be typical for bigger nanoparticles, which are also present as a limited fraction in LLCNPs formulations. The obtained results are in agreement with work of Abdel-Bar and el Basset Sanad [67], where distinct endocytic routes were observed and these were dependent on LLCNPs size. Summarizing, it is not possible to unequivocally indicate one single mechanism of LLCNPs uptake. Such behavior could be related to sample heterogeneity, which is composed of cubosomes of various sizes and low number of accompanying lipid nanoparticles (Additional file 1: Fig. S2). Nevertheless, as the most dominant route, the energy-independent and cholesterol-dependent uptake was indicated.

## Conclusions

To conclude, this work presents the impact of GMO- and PHT-based LLCNPs on cells MSU 1.1 and HeLa at different levels: cyto- and genotoxicity, internalization pathways and ROS generation. PHT-based LLCNPs appear to be much more toxic than GMO ones, what was observed in different results for metabolic activity, reactive oxygen species generation and cytoskeleton disruption. In comparison, PHT-based LLCNPs exhibit no cytotoxic effect up to 13 µg/ml, while GMO-based LLCNPs emerge cytotoxic above concentration of 100 µg/ml. Finally, this work provides evidence that GMO- and PHT-based LLCNPs have impact also on the expression of genes, involved in pathways of DNA damage and repair, mitochondrial function and proliferation. Taking these results into account, GMO-based LLCNPs emerge as potentially more feasible candidates for drug delivery systems, as their impact on cells is not as harmful as that of PHT-based ones. Moreover, they were internalized by cells monolayers and 3D spheroids efficiently, mostly by the energy-independent and cholesterol-dependent pathway into perinuclear localization inside cytoplasm, which makes them interesting material for drug delivery systems.

## Methods

### Preparation of LLCNPs

**Fig. 8:**
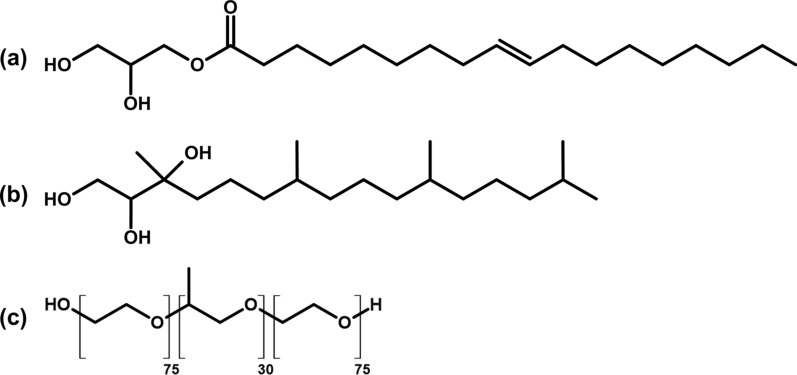
Chemical structure of **a** glyceryl monooleate, **b** phytantriol and **c** Pluronic F127

LLCNPs were prepared using the well-known top-down approach [68]. The glyceryl monooleate (GMO, 90%, IOI Olea GmbH, Hamburg, Germany) and phytantriol (PHT, DSM, Netherlands) were used as a structure-forming lipid and Pluronic F-127 (Merck, Darmstadt, Germany) as a surfactant stabilizing the LLCNPs aqueous colloid (dispersion). The chemical structures of LLCNPs components are presented in Fig. 8. Considering the temperature-composition phase diagrams [38, 69] two different compositions for each lipid were chosen, namely 2 and 20 w/w% of the lipid. The samples will be further denoted as GMO 2%, GMO 20%, and PHT 2%, PHT 20% indicating the w/w% of the lipid. The Pluronic F-127 concentration was kept constant for each composition at 0.5 w/w%. The bulk cubic phase was prepared by weighing the appropriate amount of GMO or PHT and Pluronic F-127 into a vial and melting them together at 40 °C until the flowing viscous liquid was obtained. Then this melted mixture was added with an appropriate amount of deionized water (H_2_O_d_, 19 MΩ × cm Milli-Q) at 40 °C, shortly stirred and left for 24 h for the hydration. To obtain the dispersion of LLCNPs, the hydrated bulk cubic phase was homogenized by probe sonicator (Branson 250) at output power of 60 W with a 2 s-ON and 2 s-OFF mode for a total 15 min, until milky dispersion was formed. Prior further analyses LLCNPs dispersions were left for the stabilization at RT for 24 h.

For the uptake studies, LLCNPs were fluorescently labelled with Nile Red (Merck, Darmstadt, Germany). For this purpose, the stage of the bulk cubic phase was completed by the addition of the Nile Red solution in ethanol (100 µl of 1 mg/ml stock NR solution) (99.8%, Avantor Performance Materials Poland S.A., Gliwice, Poland) to the melted mixture of the lipid and Pluronic F-127. The formed solution was further vacuum dried until the lipid film was formed on the bottom of the vial. Further steps of preparation of LLCNPs dispersion were similar as described above. Unattached Nile Red molecules were removed by the ultrafiltration centrifugation with Amicon® Ultra-4 Centrifugal Filter Devices (10,000 MWCO) according to manufacturer’s instructions. Briefly, 250 μl stained LLCNPs were added onto the filter membrane and centrifuged at 3000 g for 30 min. Fluorescently labelled nanoparticles were recovered from the filter and re-dispersed in the deionized water up to the initial volume.

### Cryogenic transmission electron microscopy

Specimens dedicated for cryogenic transmission electron microscopy (cryo-TEM) were vitrified using Cryoplunge 3 System at humidity ca. 95% and in room temperature. Briefly, a droplet of nanoparticles suspension was deposited on a perforated carbon-coated copper grid (Lacey C only, Ted Pella Inc., CA, United States) and plunged into a liquid ethane. Afterwards as prepared specimens were transferred to the 626 Gatan Cryo-holder (Gatan, CA, United States) and imaged using Jeol JEM-1400 TEM maintaining low temperature about −175 °C during the observation.

### Small Angle X-ray scattering

Small angle X-rays scattering measurements of the cubosomes samples were conducted using a SAXS/WAXS XEUSS 2.0 system (XENOCS, Grenoble, France). The system is equipped in MetalJet microfocus X-ray source (λ = 0.134 nm) with a liquid metal (gallium/indium alloy) (Excillum AB, Kista, Sweden), PILATUS 3 R 1 M hybrid photon counting detector (Dectris AG, Baden, Switzerland) and Fox 3D Ga ultra-low divergence mirrors. The sample-to-detector distance was 2535 mm, which in the high-resolution mode of X-ray optics, covers the scattering vector range 0.06 < *s* < 2.5 nm^−1^. Measurements were conducted at room temperature stabilized using the Linkam temperature attachment (Linkam Scientific Instruments Ltd., Waterfield, UK) and borosilicate glass capillaries (Hilgenberg GmbH, Malsfeld, Germany). For each sample, 4 frames (1800s/frame) were collected. Then the collected frames were processed using FOXTROT [70], and the buffer scattering was subtracted using PRIMUS [71].

### Particles size distribution and zeta potential

The particle size distribution (PSD) and zeta potential (ζ) of prepared LLCNPs, as well as their stability over time, were measured on Zetasizer Nano ZS (Malvern Instruments Ltd., Malvern, UK), based on the non-invasive dynamic light scattering method (DLS-NIBS) using an angle of 173° and electrophoretic light scattering (ELS), respectively. Measurements were performed for diluted LLCNPs dispersion at 25 °C, and results are the mean ± standard deviation (SD) of three analyses.

### Cell culture

Human cervical cancer cell line (HeLa) obtained from the American Type Culture Collection (ATCC, Virginia, United States) and human fibroblast cell line (MSU 1.1) obtained from Prof. C. Kieda (CBM, CNRS, Orléans, France) were used for in vitro studies. Cells were cultured in a complete medium (Dulbecco’s Modified Eagle’s Medium, DMEM) supplemented with 10% fetal bovine serum (FBS), 100 units/ml penicillin, 100 μg/ml streptomycin and maintained at 37 °C in humidified atmosphere containing 5% CO_2_.

### WST-1 Assay

MSU 1.1 and HeLa cells were cultured in 96–well plates in an amount of 6 × 10^3^ cells per well. After 24 h cells were treated for diverse periods of time with different LLCNPs concentration (w/w of lipid): for GMO in concentration range from 1 µg/ml to 200 µg/ml and for PHT in concentration range from 0.8125 µg/ml to 22 µg/ml. Subsequently, the WST–1 reagent was administered to each well and further incubated for 1.5 h. Next the absorbance was measured using a plate reader (Anthos Zenyth 340rt) at 450 nm wavelength (with a reference at 620 nm). The relative cell viability (%) was expressed as a percentage relative to the negative control. Data are reported as the average ± standard deviation (SD) of experiments performed in triplicate.

To extend cytotoxicity studies, the impact of LLCNPs on HeLa 3D spheroids were evaluated. 1500 cells were seeded on GravityTRAP™ ULA plates (inSphero, Schlieren, Switzerland), centrifuged for 2 min at 250 RCF and cultured for 48 h until constitution of spheroids. After that, spheroids were administered with different concentrations of LLCNPs for 24 h. Thereafter, spheroids were rinsed with PBS and transferred to new 96-well plate in the amount of 3 spheroids per well. As previously, to each well WST-1 reagent was added and after 1.5 h of incubation, the absorbance at 450 nm was measured.

### Intracellular ROS generation

For the quantitative evaluation of hydrogen peroxide activity, cells were cultured in 96-well plates in an amount of 6 × 10^3^ cells per well. After 24-h incubation, cells were washed with Hank’s Balanced Salt Solution (HBSS) buffer and treated with 100 µM DCFH-DA (Sigma Aldrich, Missouri, United States) in HBSS solution for 30 min at 37 °C. Subsequently, cells were washed and incubated with HBSS for another 30 min. Subsequently, different concentrations of LLCNPs in the HBSS buffer were added. As a positive control, 1 mM H_2_O_2_ in HBSS were prepared. Level of generated ROS is correlated with the conversion of 2,7-dichlorodihydrofluorescein diacetate (DCFH-DA) by intracellular esterases into the polar and nonfluorescent DCFH form that is retained intracellularly, and where it is further ROS-oxidized into 2′–7′dichlorofluorescein (DCF)—a fluorescent product. The ROS generation ability is therefore expressed as a ratio of measured fluorescence intensity of the sample exposed to LLCNPs to the control without exposure to LLCNPs. Fluorescence measurements were performed on the Synergy H1 Hybrid Reader at the excitation/emission of 485 nm/530 nm. Experiments were conducted at non-toxic concentrations of LLCNPs.

For the qualitative evaluation of the intracellular reactive oxygen species (ROS) generation in vitro, the CellRox® Green Reagent (Life Technologies, California, United States) was used, according to the manufacturer’s instructions. Briefly, HeLa and MSU 1.1 cells were treated with selected concentrations of LLCNPs for 3 h. Menadione (30 μM) was used as a positive control. Afterward, cells were added with the CellRox® at final concentration of 5 μM) for 30 min at 37 °C, stained with nuclear fluorescent dye Hoechst 33,342, and observed under confocal laser scanning microscopy (CLSM, Olympus FV1000).

### Cytoskeleton integrity evaluation

Approximately 1 × 10^4^ cells per well were seeded into 8–well Lab-Tek dish. After treatment with different concentrations of LLCNPs, cells were fixed with 3.7% formaldehyde (Polysciences, Pennsylvania, United States) and incubated in 0.1% Triton X–100 (Merck, Darmstadt, Germany) to allow permeabilization. Subsequently, 1% BSA was added to each well and incubated for 20 min. Respectively, 0.025% phalloidin (Invitrogen, California, United States) in 1% BSA were distributed into each well and incubated for 20 min. Finally, nuclei were stained with Hoechst 33,342 solution. Stained cells were observed using a confocal laser scanning microscope (CLSM, FV1000, Olympus).

### Genes expression studies

For gene expression studies cells were cultured in 6-well plates in the amount of 1.8 × 10^5^ cells per well. Following that, cells were treated with LLCNPs and RNA was isolated, using the RNeasy Mini Kit (Qiagen, Hilden, Germany), according to the manufacturer’s instruction. The purified RNA was eluted in RNA-free water and analyzed spectrophotometrically (NanoDrop 2000, Thermo Fisher Scientific, Massachusetts, United States). The cDNA samples for RT-PCR experiments were synthesized from 1 μg of total RNA and anchored-oligo(dT)_18_ primers, using the RevertAid RT Reverse Transcription Kit (Thermo Fisher Scientific Massachusetts, United States). During studies, the expression of *ACTB*, *DHFR, CDK1, GADD45A1* and *GAPDH* genes was investigated. The primers for investigated genes were designed or selected based on earlier obtained results by Atha et al. [72] *GAPDH* was selected as a reference gene to normalize other gene expression values. The real-time amplification reactions with the SYBR Green detection chemistry were run in triplicate using 96-wells plates and the iCycler CFX98 thermocycler (Bio-Rad, California, United States). In addition, for each reaction, a calibration curve was determined using each primer pair and selected cDNA as a template, in serial diminishing copies number from 10^9^ to 10^4^ copies. Primers used in real-time RT-PCR are shown in Additional file 1: Table S2. The conditions were set as follows: initial denaturation step of 95 °C for 10 min, followed by 40 cycles of denaturation at 95 °C for 45 s, annealing at 60 °C for 30 s, and extension at 72 °C for 40 s. The amplification process was followed by a melting curve analysis, from 60 °C to 95 °C, with temperature increasing steps of 0.5 °C every 10 s. Baseline and threshold cycles (Ct) were automatically determined using the Bio-Rad iQ Software 3.0. The reaction results were recorded and analyzed using Chromo4™ System software.

### Internalization pathways

To block energy-dependent mechanisms of LLCNPs uptake, the grown HeLa and MSU 1.1 cells were incubated at 4 °C for 1 h. Media was then replaced with cold serum-free DMEM containing 25 μg/ml of Nile Red stained LLCNPs and incubated for another 1 h at 4 °C. Serum-free DMEM was used to exclude the effect of protein adsorption on NPs surfaces that can potentially alter the endocytic pathways. Afterward, cells were rinsed with PBS, maintained in phenol red-free medium (Fluoro Bright DMEM, Gibco), and imaged using a laser scanning confocal microscope (CLSM, Olympus FV1000).

The influence of different endocytic inhibitors on the cellular uptake of LLCNPs was also assessed. Briefly, the seeded cells were incubated separately with methyl-β-cyclodextrin (2.5 mg/ml), as an inhibitor of caveolae/lipid raft-dependent endocytosis; chlorpromazine hydrochloride (5 μg/ml), as an inhibitor of clathrin-mediated endocytosis; and wortmannin (150 ng/ml), as macropinocytosis inhibitor, nocodazole (2.5 μg/ml), as inhibitor of microtubule polymerization cytochalasin D (5 μg/ml), as inhibitor of F-actin polymerization (for 1 h at 37 °C). Subsequently, cells were incubated with 25 μg/ml of Nile Red stained LLCNPs for 1 h and imaged as mentioned above.

### Statistical analysis

All experiments were done in triplicate, and the results were presented as mean ± standard deviation. The experimental data were analyzed by ANOVA with post-hoc Tukey HSD test. Statistical significance was marked with asterisks depending on the p-value: *—p ≤ 0.05, **—p ≤ 0.01, ***—p ≤ 0.001, ****—p ≤ 0.0001.

## Supplementary Information

**Additional file 1.** Additional figures and tables.

## Data Availability

Not applicable.
