# Supplementary material for: Comprehensive and comparative studies on nanocytotoxicity of glyceryl monooleate- and phytantriol-based lipid liquid crystalline nanoparticles
Source: J Nanobiotechnology. 2021 Jun 3;19:168. doi: 10.1186/s12951-021-00913-5 (PMC8176590; doi:10.1186/s12951-021-00913-5)
Supplement: Supplementary file 1 — Additional file 1. Additional figures and tables. [file 12951_2021_913_MOESM1_ESM.docx]

**Supplementary materials**

**Comprehensive and comparative studies on nanocytotoxicity of glyceryl monooleate- and phytantriol-based lipid liquid crystalline nanoparticles**

**Jakub Jagielski^1^, Łucja Przysiecka^1^, Dorota Flak^1^, Magdalena Diak^1^, Zuzanna Pietralik-Molińska^2^, Maciej Kozak^2^, Stefan Jurga^1^, Grzegorz Nowaczyk^1 *^**

**^1^NanoBioMedical Centre, Adam Mickiewicz University, Wszechnicy Piastowskiej 3, 61-614 Poznań, Poland**

**^2^Department of Macromolecular Physics, Faculty of Physics, Adam Mickiewicz University, Uniwersytetu Poznańskiego 2, 61-614 Poznań, Poland**

**^*^ Corresponding author, email: grzegorz.nowaczyk@amu.edu.pl**

Tab. S1. Statistically significant differences in viability of HeLa and MSU 1.1 cells incubated with LLCNPs to control after 3, 24, 48, 72 hours.

***3 h incubation***

| **MSU 1.1 GMO 2%** | |
| --- | --- |
| **Concentration** | **P value** |
| 200 µg/ml | ≤ 0.01 |
| **MSU 1.1 PHT 2%** | |
| **Concentration** | **P value** |
| 22 µg/ml | ≤ 0.05 |
| 6,5 µg/ml | ≤ 0.01 |
| 3,25 µg/ml | ≤ 0.05 |
| **MSU 1.1 PHT 20%** | |
| **Concentration** | **P value** |
| 22 µg/ml | ≤ 0.001 |
| 20 µg/ml | ≤ 0.001 |
| 18 µg/ml | ≤ 0.01 |
| 6,5 µg/ml | ≤ 0.001 |
| 3,25 µg/ml | ≤ 0.001 |
| 1,625 µg/ml | ≤ 0.05 |
| **HeLa GMO 2%** | |
| **Concentration** | **P value** |
| 200 µg/ml | ≤ 0.001 |
| 150 µg/ml | ≤ 0.001 |
| 100 µg/ml | ≤ 0.001 |
| 1 µg/ml | ≤ 0.05 |
| **HeLa GMO 20%** | |
| **Concentration** | **P value** |
| 200 µg/ml | ≤ 0.001 |
| 50 µg/ml | ≤ 0.01 |
| 20 µg/ml | ≤ 0.01 |
| 10 µg/ml | ≤ 0.05 |
| 1 µg/ml | ≤ 0.05 |

**24 h incubation**

| **HeLa PHT 2%** | |
| --- | --- |
| **Concentration** | **P value** |
| 22 µg/ml | ≤ 0.001 |
| 20 µg/ml | ≤ 0.001 |
| 18 µg/ml | ≤ 0.001 |
| 13 µg/ml | ≤ 0.001 |
| 6,5 µg/ml | ≤ 0.001 |
| 3,25 µg/ml | ≤ 0.001 |
| 1,625 µg/ml | ≤ 0.001 |
| 0,8125 µg/ml | ≤ 0.001 |
| **HeLa PHT 20%** | |
| **Concentration** | **P value** |
| 22 µg/ml | ≤ 0.001 |
| 20 µg/ml | ≤ 0.001 |
| 18 µg/ml | ≤ 0.001 |
| 13 µg/ml | ≤ 0.001 |
| 6,5 µg/ml | ≤ 0.001 |
| 3,25 µg/ml | ≤ 0.01 |
| 1,625 µg/ml | ≤ 0.05 |
| 0,8125 µg/ml | ≤ 0.01 |
| **MSU 1.1 PHT 2%** | |
| **Concentration vs control** | **P value** |
| 22 µg/ml | ≤ 0.001 |
| 20 µg/ml | ≤ 0.001 |
| 18 µg/ml | ≤ 0.001 |
| **MSU 1.1 PHT 20%** | |
| **Concentration** | **P value** |
| 22 µg/ml | ≤ 0.001 |
| 20 µg/ml | ≤ 0.001 |
| 18 µg/ml | ≤ 0.001 |
| **HeLa GMO 2%** | |
| **Concentration** | **P value** |
| 200 µg/ml | ≤ 0.001 |
| 150 µg/ml | ≤ 0.001 |
| 100 µg/ml | ≤ 0.001 |
| 50 µg/ml | ≤ 0.05 |
| 20 µg/ml | ≤ 0.05 |

| **HeLa GMO 20%** | |
| --- | --- |
| **Concentration** | **P value** |
| 200 µg/ml | ≤ 0.001 |
| 150 µg/ml | ≤ 0.001 |
| 100 µg/ml | ≤ 0.01 |
| 50 µg/ml | ≤ 0.05 |
| **MSU 1.1 GMO 2%** | |
| **Concentration** | **P value** |
| 200 µg/ml | ≤ 0.001 |
| 150 µg/ml | ≤ 0.001 |
| 100 µg/ml | ≤ 0.001 |
| 50 µg/ml | ≤ 0.01 |
| 20 µg/ml | ≤ 0.05 |
| 10 µg/ml | ≤ 0.05 |
| 1 µg/ml | ≤ 0.05 |
| **MSU 1.1 GMO 20%** | |
| **Concentration** | **P value** |
| 200 µg/ml | ≤ 0.001 |
| 150 µg/ml | ≤ 0.001 |

**48 h incubation**

| **HeLa PHT 2%** | |
| --- | --- |
| **Concentration** | **P value** |
| 22 µg/ml | ≤ 0.001 |
| 20 µg/ml | ≤ 0.001 |
| 18 µg/ml | ≤ 0.01 |
| 13 µg/ml | ≤ 0.01 |
| 6,5 µg/ml | ≤ 0.05 |
| 3,25 µg/ml | ≤ 0.05 |
| **HeLa PHT 20%** | |
| **Concentration** | **P value** |
| 22 µg/ml | ≤ 0.001 |
| 20 µg/ml | ≤ 0.001 |
| 18 µg/ml | ≤ 0.001 |
| **MSU 1.1 PHT 2%** | |
| **Concentration** | **P value** |
| 22 µg/ml | ≤ 0.001 |
| **MSU 1.1 PHT 20%** | |
| **Concentration** | **P value** |
| 22 µg/ml | ≤ 0.001 |
| 20 µg/ml | ≤ 0.001 |
| 18 µg/ml | ≤ 0.001 |
| 13 µg/ml | ≤ 0.001 |
| 6,5 µg/ml | ≤ 0.001 |
| 3,25 µg/ml | ≤ 0.001 |
| 1,625 µg/ml | ≤ 0.001 |
| 0,8125 µg/ml | ≤ 0.01 |
| **HeLa GMO 2%** | |
| **Concentration** | **P value** |
| 200 µg/ml | ≤ 0.001 |
| 150 µg/ml | ≤ 0.001 |
| **HeLa GMO 20%** | |
| **Concentration** | **P value** |
| 200 µg/ml | ≤ 0.001 |
| **MSU 1.1 GMO 2%** | |
| **Concentration** | **P value** |
| 200 µg/ml | ≤ 0.001 |
| 150 µg/ml | ≤ 0.001 |
| 100 µg/ml | ≤ 0.001 |
| 50 µg/ml | ≤ 0.05 |
| 20 µg/ml | ≤ 0.05 |
| 10 µg/ml | ≤ 0.01 |
| 5 µg/ml | ≤ 0.05 |
| **MSU 1.1 GMO 20%** | |
| **Concentration** | **P value** |
| 200 µg/ml | ≤ 0.001 |
| 150 µg/ml | ≤ 0.001 |
| 100 µg/ml | ≤ 0.05 |
| 50 µg/ml | ≤ 0.00 |
| 20 µg/ml | ≤ 0.00 |
| 10 µg/ml | ≤ 0.001 |
| 5 µg/ml | ≤ 0.001 |

**72 h incubation**

| **HeLa PHT 2%** | |
| --- | --- |
| **Concentration** | **P value** |
| 22 µg/ml | ≤ 0.001 |
| **HeLa PHT 20%** | |
| **Concentration** | **P value** |
| 22 µg/ml | ≤ 0.001 |
| 20 µg/ml | ≤ 0.001 |
| 18 µg/ml | ≤ 0.001 |
| 13 µg/ml | ≤ 0.001 |
| 6,5 µg/ml | ≤ 0.001 |
| 3,25 µg/ml | ≤ 0.001 |
| 1,625 µg/ml | ≤ 0.001 |
| 0,8125 µg/ml | ≤ 0.05 |
| **MSU 1.1 PHT 2%** | |
| **Concentration** | **P value** |
| 22 µg/ml | ≤ 0.001 |
| 20 µg/ml | ≤ 0.001 |
| 18 µg/ml | ≤ 0.001 |
| 13 µg/ml | ≤ 0.01 |
| 6,5 µg/ml | ≤ 0.01 |
| 3,25 µg/ml | ≤ 0.05 |
| **MSU 1.1 PHT 20%** | |
| **Concentration** | **P value** |
| 22 µg/ml | ≤ 0.001 |
| 20 µg/ml | ≤ 0.001 |
| 18 µg/ml | ≤ 0.001 |
| **HeLa GMO 2%** | |
| **Concentration** | **P value** |
| 200 µg/ml | ≤ 0.001 |
| 150 µg/ml | ≤ 0.001 |
| 100 µg/ml | ≤ 0.001 |
| 50 µg/ml | ≤ 0.01 |
| 20 µg/ml | ≤ 0.05 |
| **HeLa GMO 20%** | |
| **Concentration** | **P value** |
| 200 µg/ml | ≤ 0.001 |
| 150 µg/ml | ≤ 0.001 |
| 100 µg/ml | ≤ 0.001 |
| 50 µg/ml | ≤ 0.001 |
| 20 µg/ml | ≤ 0.001 |
| 10 µg/ml | ≤ 0.001 |
| **MSU 1.1 GMO 2%** | |
| **Concentration** | **P value** |
| 200 µg/ml | ≤ 0.001 |
| 150 µg/ml | ≤ 0.001 |
| 100 µg/ml | ≤ 0.001 |
| 50 µg/ml | ≤ 0.01 |
| 20 µg/ml | ≤ 0.01 |
| 10 µg/ml | ≤ 0.01 |
| **MSU 1.1 GMO 20%** | |
| **Concentration** | **P value** |
| 200 µg/ml | ≤ 0.05 |
| 150 µg/ml | ≤ 0.01 |
| 100 µg/ml | ≤ 0.05 |
| 50 µg/ml | ≤ 0.05 |
| 20 µg/ml | ≤ 0.05 |
| 10 µg/ml | ≤ 0.05 |

Tab. S2. Primers for gene expression evaluation.

| CDK1 | F: CTTGGCTTCAAAGCTGGCTC |
| --- | --- |
|  | R: GGGTATGGTAGATCCCGGCT |
| DHFR | F: GCTGCTGTCATGGTTGGTTC |
|  | R: GAGGTTGTGGTCATTCTCTGGA |
| ACTB | F: TCACCC ACACTG TGCCCATCTACGA |
|  | R: AGCGGAACCGCTCATTGCCAATGG |
| GADD45A1 | F: AGCTGCTCAACGTAATCCACA |
|  | R: TCATTCAGATGCCATCACCGT |
| GAPDH | F: AAGGTCGGAGTCAACGGATTT |
|  | R: ACCAGAGTTAAAAGCAGCCCTG |


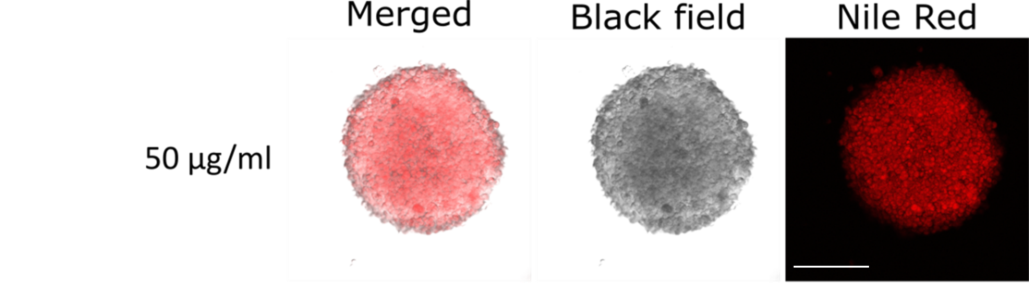


Fig. S1. CLSM images of HeLa spheroids after 24 h incubation with 50 μg/ml Nile Red stained GMO 2% LLCNPs. Scale bar: 100 µm.


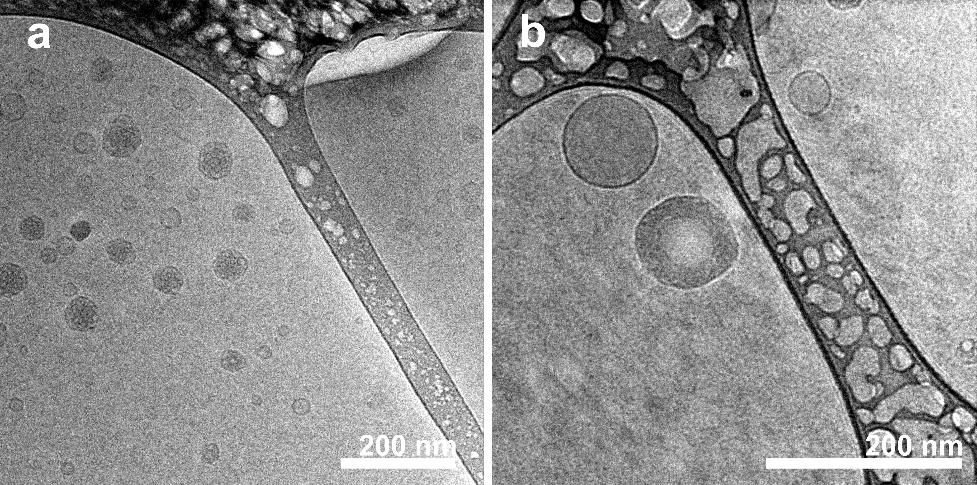


Fig. S2. Cryo-TEM images of alternative forms of LLCNPs like liposomes or partially formed cubosomes - (a) GMO 2% and (b) PHT 2%.
